# Use of a fluoroscopy-based robotic-assisted total hip arthroplasty system produced greater improvements in patient-reported outcomes at one year compared to manual, fluoroscopic-assisted technique

**DOI:** 10.1007/s00402-024-05230-8

**Published:** 2024-02-24

**Authors:** Graham B.J. Buchan, Christian B. Ong, Christian J. Hecht II, Charles A. DeCook, Luke S. Spencer-Gardner, Atul F. Kamath

**Affiliations:** 1 Department of Orthopaedic Surgery, Orthopaedic and Rheumatologic Institute, Cleveland Clinic Foundation, 9500 Euclid Avenue, Cleveland, OH 44195 USA; 2 Arthritis and Total Joint Specialists, 2000 Howard Farm Drive, Suite 200, Cumming, GA 30041 USA; 3 Department of Orthopaedic Surgery, Mayo Clinic, 4500 San Pablo Rd S, Jacksonville, FL 32224 USA

**Keywords:** Arthroplasty, Hip, Robotic-assisted surgery, Patient-reported outcome measures

## Abstract

**Introduction:**

The adoption of new technology should be supported by improvements in patient-reported outcomes (PROMs). The purpose of this study was to assess the one-year PROMs of patients who underwent total hip arthroplasty (THA) using a novel, fluoroscopy-based, robotic-assisted (RA-THA) system when compared to a manual, fluoroscopic-assisted technique (mTHA).

**Materials and methods:**

A review of 91 consecutive mTHA and 85 consecutive RA-THA via a direct anterior approach was conducted. All cases were performed by the same surgeon at the same institution, for a pre-operative diagnosis of osteoarthritis, avascular necrosis, or rheumatoid arthritis. Outcomes included one-year Veterans RAND-12 (VR-12) Physical/Mental, Hip Disability and Osteoarthritis Outcome (HOOS) Pain/Physical Function/Joint Replacement, and University of California Los Angeles (UCLA) Activity scores, as well as the difference between pre-operative and one-year post-operative PROMs.

**Results:**

Patients in the RA-THA cohort had lower pre-operative HOOS-JR scores compared to patients in the mTHA cohort (37.0 vs. 43.1; *p* = 0.031). Cohorts experienced similar one-year post-operative VR-12, HOOS, and UCLA Activity scores. Patients in the RA-THA cohort experienced greater improvements across all pre- and post-operative HOOS scores compared to patients in the mTHA cohort: Pain (+ 54.7 vs. +42.1; *p* = 0.009), Physical Function (-41.6 vs. -28.7; *p* = 0.007), and Joint Replacement (+ 46.6 vs. +33.0; *p* = 0.002). These differences exceeded minimum clinically important difference (MCID).

**Conclusions:**

Both manual and robotic cohorts experienced benefit from THA at one-year post-operative. Importantly, the use of a novel, fluoroscopy-based robotic assistance system for primary THA resulted in greater improvements in PROMs at one-year relative to manual technique.

**Supplementary Information:**

The online version contains supplementary material available at 10.1007/s00402-024-05230-8.

## Introduction

Total hip arthroplasty (THA) is the standard treatment for end-stage osteoarthritis of the hip, providing a reduction in patient pain while also improving mobility [1–4]. However, up to 27% of THA patients report having unfulfilled expectations regarding their surgery [1, 5, 6], with dissatisfaction often being driven by post-operative complications and poor functional outcomes [1]. Imprecise peri-operative acetabular cup positioning has been implicated as a driver of poor outcomes following THA [7], encouraging the use of additional intra-operative assistance to ensure favorable component alignment.

The use of robotic-assistance for total hip arthroplasty (RA-THA) has seen increased popularity over the past decade [8]. By providing intra-operative guidance and mechanical assistance to the surgeon, these systems have been associated with improved acetabular cup placement accuracy and precision [9, 10], reduced dislocation rates [11], and shortened hospital length of stay [12], compared to manual unassisted THA (mTHA). In addition, some studies have suggested that RA-THA improves post-operative patient-reported outcome measures (PROMs) relative to mTHA [13, 14], though conflicting evidence exist in the literature [11, 13–21].

In 2021, a novel, fluoroscopy-based RA-THA platform received approval from the United States (U.S.) Food and Drug Administration (FDA) for use in primary THA. Prior investigations have demonstrated that utilizing this system resulted in improved acetabular cup placement accuracy and precision, and reduced leg-length discrepancy, relative to mTHA [22, 23]. However, the short-term outcomes of patients who underwent THA with this system are not well understood. Therefore, the purpose of the present investigation was to compare PROMs of patients who underwent primary THA using the novel RA-THA system, to those who underwent mTHA at one-year post-operative. We hypothesized that patients who underwent RA-THA would experience improved PROMs at one-year post-operative compared to patients who underwent mTHA.

## Materials and methods

### Study design

Institutional Review Board approval was obtained prior to the initiation of this study. We performed a retrospective cohort analysis on a consecutive series of patients who received manual, fluoroscopy-assisted THA (mTHA) and fluoroscopy-based RA-THA at our institution from the primary study surgeon between 2021 and 2022. Our primary outcome of interest was the change in patient PROM scores from pre-operative baseline to 1-year following surgery. Patient PROM scores were collected during pre-operative visits and one-year post-operative follow-up office visits as a part of standard institutional practice, and were extracted from the electronic health record [24].

Inclusion criteria for this study were patients ≥ 18 years of age who underwent primary unilateral direct anterior approach (DAA) THA by the primary surgeon. Exclusion criteria for this study included patients who underwent THA for a femoral neck fracture, revision THA, bilateral THA, and patients < 18 years of age. Based on the previously reported Minimum Clinically Important Difference (MCID) for HOOS JR, we sought to include approximately 60 patients per treatment arm to detect an 18-point difference in HOOS JR scores, with 80% statistical power [25].

### Surgical technique

**Fig. 1:**
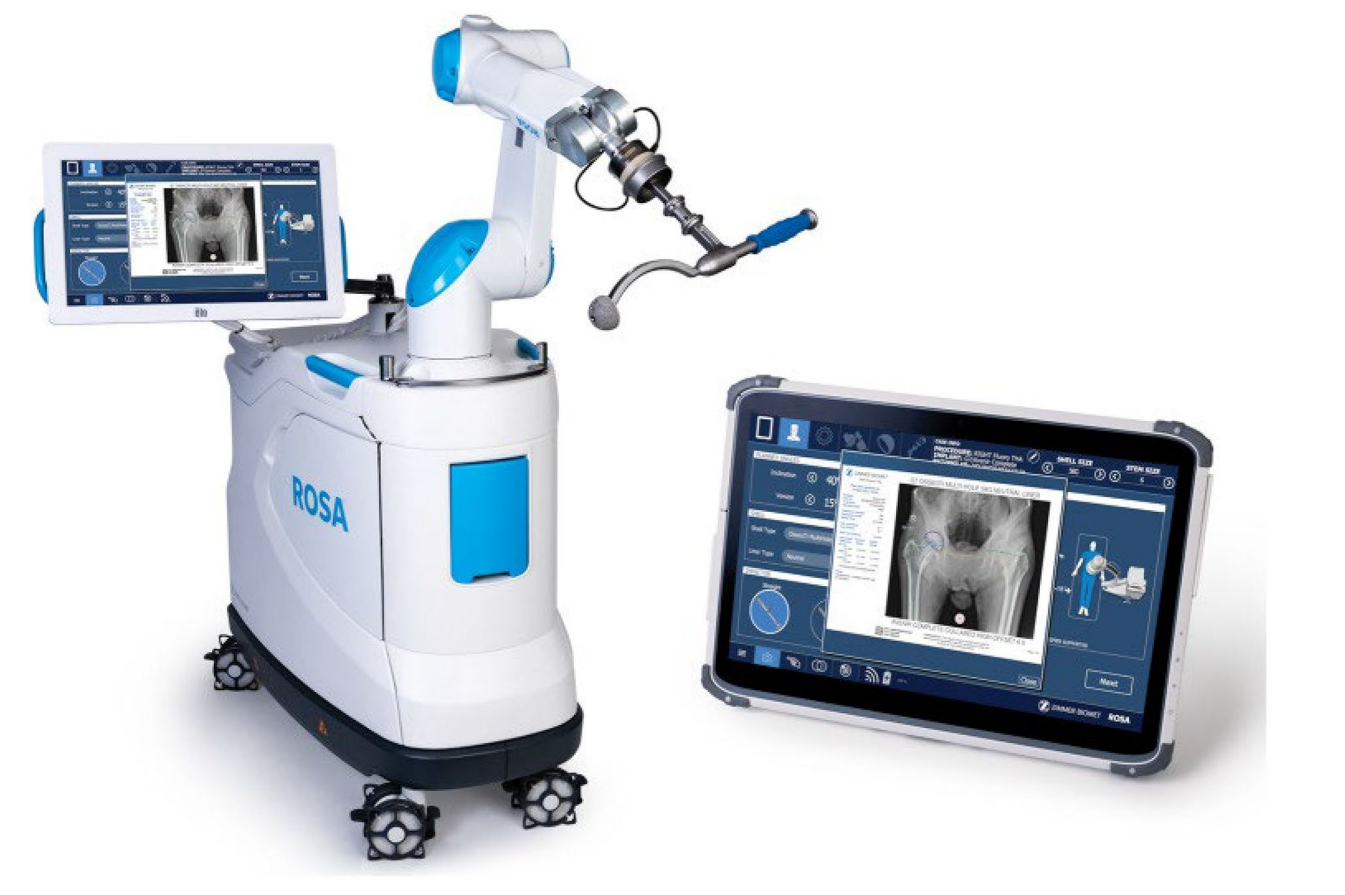
An overview of the ROSA® Hip System. Deployment of this system includes the following steps: (1) Connecting the tablet to the robotic unit using Wi-Fi; (2) Selecting surgical parameters including planned cup angles, measurements, shell and stem type, impactor and C-arm diameter; (3) Installing the quick connect interface at the end of the robotic arm; (4) Draping the robotic arm and robotic unit; and (5) Calibrating the force sensor

**Fig. 2:**
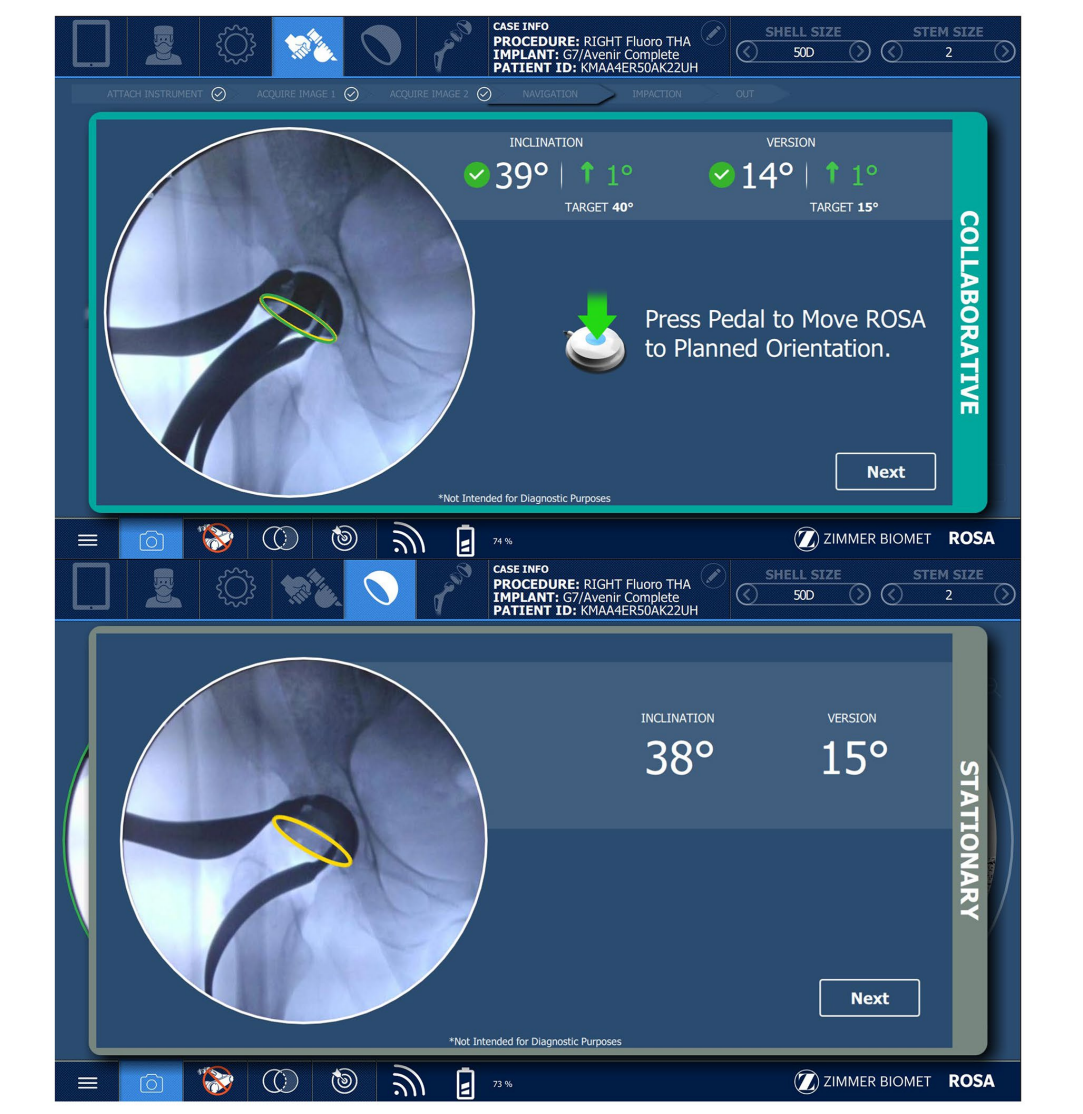
An overview of the intra-operative navigation interface of the ROSA® Hip System. (Top) Screenshot of Cup Impaction panel. (Bottom) Screenshot of Cup Verification panel

For the study cohort, a consecutive series of patients underwent DAA-RA-THA between September 2021 and July 2022 with the assistance of a fluoroscopy-based robotic platform, the ROSA® Total Hip System (Zimmer CAS, Montreal, Canada), using a surgical workflow previously detailed by Kamath et al. [23]. An overview of the ROSA® robotic system is provided in Fig. 1, along with screenshots from the intra-operative navigation workflow in Fig. 2. The control cohort consisted of a consecutive series of patients who underwent manual DAA-mTHA just prior to September 2021, for which the principal surgeon employed fluoroscopic guidance using a standard 12-inch C-arm to assist with leveling the pelvis, bone preparation, and the assessment of component position. Other than the intra-operative use of the robotic platform, pre-operative evaluation, surgical technique, and post-operative workflow were identical for both cohorts.

### PROM collection instruments

Patient-reported outcome measures were collected using previously validated and standardized instruments as described below:

- Veterans RAND 12 (VR-12) Physical (PCS) and Mental (MCS) Component scores: Twelve-item questionnaire assessing disease burden in terms of detriment to Physical and Mental Health-Related Quality of Life (HRQoL). Raw scores were standardized to population T-scores ranging from 0 (worst HRQoL) to 100 (best HRQoL) [26].
- Hip Dysfunction and Osteoarthritis Outcome Score (HOOS) (Pain, Physical Function (PS), and Joint replacement (JR) scores): Forty-item questionnaire assessing hip disability and functional outcomes, further subdivided into categories pertaining to Pain, PS and JR [27]. Raw scores were standardized to interval scores ranging from 0 (total hip disability) to 100 (perfect hip health) [28].
- University of California, Los Angeles (UCLA) Activity Scale score: Ten-item questionnaire assessing physical activity level for individuals undergoing total joint arthroplasty (TJA), with scores ranging from 1 (lowest activity) to 10 (highest activity) [29].

### Radiographic analysis

Acetabular component placement was also assessed to better understand the results of our outcome metrics. Acetabular cup orientation was determined by analyzing postoperative anteroposterior (AP) pelvic radiographs using Martell *Hip Analysis Suite Software* (*version* 8.0.4.5., *Martell Hip Analysis Suite*™, Chicago, IL). Radiographs used for analysis were standardized to standing AP pelvis studies obtained at routine 6-week postoperative follow-up visits. The measurements were used to determine if the acetabular cups were positioned within the Lewinnek safe zone, defined as 40 ± 10˚ of cup inclination and 15 ± 10˚ of cup anteversion [30]. All patients in the study had a pre-operative goal of 40˚/15˚ cup inclination and anteversion.

### Statistical analysis

A comparison of treatment data and pre-operative patient demographics between study cohorts was performed. Changes in PROM scores from baseline to one-year post-operative were calculated for each individual patient who completed both surveys. The average change in PROM scores was also calculated for each cohort and additionally stratified based on acetabular cup position in the Lewinnek safe zone. Continuous variables were reported as means and standard deviations (SD) and compared using independent samples t-tests. Categorical variables were presented as frequencies and compared using Pearson’s chi-squared tests or Fisher’s exact tests when appropriate. All statistical analyses were performed using JMP Version 16.2. (SAS Institute Inc., Cary, NC, 1989–2021).

## Results

A total of 176 patients, including 91 mTHA and 85 RA-THA, were identified in the study period who met selection criteria and completed baseline PROM surveys. Comparison of baseline treatment and demographics variables demonstrated no significant differences between treatment groups (Table 1).

**Table 1:** Patient demographic and treatment data between manual THA and robotic-assisted THA cohorts

		Technique		*p*-value
		Manual THA	Robotic THA	
		*n* = 91	*n* = 85	
Age at Surgery (Years)		58.7 (15.0)	59.9 (13.7)	0.596
Gender (% Female)		55.0	44.0	0.175
Body Mass Index (BMI)		28.9 (5.3)	29.9 (4.8)	0.221
Race				0.872
(% Caucasian)		80.2	81.2	
(% Black)		19.8	18.8	
Side (% Left)		36.3	44.7	0.254
Pre-operative Diagnosis				
(% Osteoarthritis)		83.5	84.7	
(% Avascular Necrosis)		16.5	14.1	0.678
(% Rheumatoid Arthritis)		0.0	1.2	
ASA Score				0.076
(% Class I)		2.2	0.0	
(% Class II)		49.4	45.9	
(% Class III)		44.0	54.1	
(% Class IV)		4.4	0.0	

Categorical variables expressed as percentages; quantitative variables expressed as mean (SD). Significance set at a level of *p* < 0.05

The only significant difference in pre-operative PROMs was in HOOS JR scores, with the RA-THA cohort having lower average reported scores than the mTHA cohort (37.0 vs. 43.1; *p* = 0.031). All other baseline PROM scores were similar between treatment groups (Table 2). Approximately 72% of patients, 66 mTHA and 61 RA-THA, completed one-year follow-up PROM surveys. No difference was seen in post-operative VR-12, HOOS, and UCLA Activity scores when the average post-operative PROM scores were compared between cohorts (Table 2).

**Table 2:** A comparison of pre- and post-operative patient-reported outcome measure (PROM) scores between cohorts

		Treatment		
		Manual THA	Robotic THA	*p*-value
Pre-operative		*n* = 91	*n* = 85	
VR-12 PCS		27.5 (8.8)	26.3 (9.2)	0.384
VR-12 MCS		47.6 (13.7)	48.2 (13.1)	0.772
HOOS Pain		37.4 (19.6)	31.7 (20.9)	0.070
HOOS-PS		48.1 (22.9)	53.9 (23.4)	0.095
HOOS-JR		43.1 (17.7)	37.0 (19.4)	**0.031**
UCLA Activity		3.7 (1.9)	3.8 (2.0)	0.759
Post-operative		*n* = 66	*n* = 61	
VR-12 PCS		44.2 (10.1)	45.4 (11.2)	0.527
VR-12 MCS		51.3 (10.1)	50.3 (12.5)	0.646
HOOS Pain		83.5 (20.7)	84.0 (22.2)	0.897
HOOS-PS		14.4 (18.3)	12.4 (18.8)	0.555
HOOS-JR		81.0 (19.8)	83.9 (19.3)	0.444
UCLA Activity		5.2 (2.2)	5.5 (2.2)	0.432

Quantitative variables expressed as mean (SD). Significance bolded at a level of *p* < 0.05

When the average changes in post-operative PROM scores from baseline were compared, patients in the RA-THA cohort experienced greater improvements between pre- and post-operative HOOS scores compared to patients in the mTHA cohort. These key HOOS outcomes included Pain (+ 54.7 vs. +42.1; *p* = 0.009), Physical Function (-41.6 vs. -28.7; *p* = 0.007), and Joint Replacement (+ 46.6 vs. +33.0; *p* = 0.002). No differences were seen in changes for VR-12 or UCLA activity scores (Table 3). When the average changes in post-operative PROM scores were stratified based on Lewinnek safe zone placement, patients outside of safe zone in the RA-THA cohort experienced greater improvements in HOOS scores compared to patients in the mTHA cohort, including Pain (+ 58.7 vs. +34.4; *p* = 0.018), Physical Function (-50.9 vs. -22.1; *p* = 0.001), and Joint Replacement (+ 54.8 vs. +24.9; *p* < 0.001). No differences were seen between patients with cup placement within safe zone (Table 3).

**Table 3:** A comparison of the change in PROM scores between pre-operative baseline and one-year post-operative

		Treatment		*p*-value
		Manual THA	Robotic THA	
		*n* = 65	*n* = 60	
VR-12 PCS		16.0 (11.4)	18.3 (12.4)	0.286
VR-12 MCS		2.7 (11.7)	0.8 (14.1)	0.418
HOOS Pain		42.1 (25.8)	54.7 (26.3)	**0.009**
HOOS-PS		-28.7 (26.9)	-41.6 (25.3)	**0.007**
HOOS-JR		33.0 (23.7)	46.6 (21.7)	**0.002**
UCLA Activity		1.5 (2.0)	1.7 (2.1)	0.481
Safe Zone = Yes		*n* = 42	*n* = 44	
VR-12 PCS		17.9 (11.8)	20.4 (9.5)	0.298
VR-12 MCS		2.9 (11.7)	2.6 (2.2)	0.928
HOOS Pain		45.4 (25.8)	52.8 (25.7)	0.187
HOOS-PS		-31.9 (29.6)	-38.2 (25.6)	0.304
HOOS-JR		37.3 (25.0)	43.9 (21.6)	0.221
UCLA Activity		1.9 (2.2)	2.0 (2.3)	0.802
Safe Zone = No		*n* = 23	*n* = 14	
VR-12 PCS		12.7 (10.1)	13.8 (18.6)	0.823
VR-12 MCS		2.7 (12.0)	0.3 (10.6)	0.547
HOOS Pain		34.4 (24.9)	58.7 (28.3)	**0.018**
HOOS-PS		-22.1 (20.5)	-50.9 (23.3)	**0.001**
HOOS-JR		24.9 (19.9)	54.8 (21.5)	**< 0.001**
UCLA Activity		0.772 (1.31)	1.08 (1.18)	0.487

Quantitative variables expressed as mean (SD). Significance bolded at a level of *p* < 0.05

## Discussion

Patient satisfaction is an increasingly important metric for assessing the outcomes of THA. With cited benefits of RA-THA over mTHA [9–12], use of intra-operative robotics should be supported by improvements in post-operative PROMs. However, the literature has shown mixed findings, with outcomes varying between differences in surgical approach, institutional practices, and robotic platforms [11, 13–21]. The results of our investigation demonstrated that the use of a novel, fluoroscopy-based robotic assistance system for DAA THA resulted in a greater improvement in all HOOS scores, relative to mTHA, from baseline to one-year post-operative. To the authors’ best knowledge, this is the first investigation which has assessed the short-term outcomes of this particular system.

The first significant finding of this study was that there were no differences in average post-operative PROMs between the mTHA and RA-THA cohorts. Considering patients in the RA-THA cohort reported a pre-operative deficit of 6.1 points on HOOS-JR compared to patients in the mTHA cohort, RA-THA may be more effective than mTHA for improving outcomes quantified by the HOOS-JR, including operative hip pain and function, at one-year post-operative [28]. These results are especially significant given that the first 12 cases in the RA-THA series were performed during the learning period of the principal surgeon with the robotic platform [31].While these findings are in agreement with those of Fontalis and Karunaratne et al. [16, 20], other authors have reported that the use of RA-THA produced improved post-operative Harris Hip, Forgotten Joint, Short Form 12, VR-12, and UCLA activity scores relative to mTHA [13, 14, 19, 21]. Interestingly, the studies that reported no differences between PROMs were over a shorter follow-up period (2–3 years) [16, 20], relative to studies that showed improvement (2–5 years) [13, 14, 19, 21]. This suggests that the PROMs of RA-THA may improve over time, which also aligns with reports that complication rates following RA-THA substantially decline after the first year following surgery [17]. As discussed in Peters et al., caution should be exercised when comparing average scores alone, in that these findings are subject to the confounding influence of variations in pre-operative PROM scores between groups [32], which are better controlled for by assessing for changes in PROMs.

The second significant finding of this study was that the RA-THA cohort experienced a greater degree of improvement between pre-operative, and one-year post-operative HOOS scores relative to the mTHA cohort. Specifically, the RA-THA cohort experienced a benefit of 12.6, 12.9, and 13.6 additional points with regards to score improvement for HOOS Pain, HOOS-PS, and HOOS JR, respectively. These improvements in PROMs may be attributable to patients in the RA-THA cohort experiencing more favorable post-operative radiographic outcomes compared to patients in the mTHA cohort. In two separate studies by Buchan and Kamath et al., it was demonstrated that the use of this RA-THA system improved acetabular cup placement accuracy and precision with respect to pre-operative planned targets, and increased the proportion of cups placed within the Lewinnek safe zone compared to mTHA [22, 23]. Patients in the RA-THA cohort reported lower pre-operative HOOS-JR scores relative to patients in the mTHA cohort, which may have also contributed to the greater score improvement observed for HOOS-JR. The HOOS is the most well-validated joint-specific PROM for THA, and scores are generally indicative of patient pain, symptom, and functional outcomes of the hip following surgery [28, 33]. While the MCID of the HOOS Pain score has not been reported, these values exceed the MCID of the HOOS-PS and HOOS-JR, with threshold estimates of 10.01 for the HOOS-PS, and 3.9 to 15 points for the HOOS-JR [25, 33, 34]. In a related study, Singh et al. reported that one-year post-operative improvements in HOOS-JR scores were greater among mTHA patients compared to RA-THA (34.53 ± 8.91 vs. 35.48 ± 9.33; *p* = 0.002), though this difference did not exceed MCID [15]. The improvement in HOOS-JR scores were substantially greater in our study, emphasizing the potential for differences in robotic platform and/or approach to impact PROMs in RA-THA. All RA-THA procedures in Singh et al. were performed with the assistance of the Stryker MAKO® Hip, and included a mixture of posterior and DAA cases [15].

Interestingly, these differences were consistent for patients with cup placement outside of the Lewinnek safe zone. The similarity in results between patients within safe zone who received RA-THA and mTHA is unsurprising, as these patients had components placed in biomechanically optimal positions and thus would likely have similarly positive outcomes. For the patients with cups placed outside of safe zone, the RA-THA group had anteversion angles which were closer on average to the target of 15˚ relative to the mTHA group, (24.6˚ vs. 26.4˚), while the average inclination for these patients was similar (45.2˚ vs. 44.9˚). Though this difference was small, it is possible that amongst cups placed outside of safe zone, patients who received RA-THA had components placed in more biomechanically optimized positions compared to patients who received mTHA. These differences could have translated into the functional improvements reflected in the PROMs.

Our study has a number of limitations. First, this was a retrospective review, which had the potential to introduce documentation biases. To mitigate risk of bias, an electronic health record system that captured patient-data from multiple care centers was utilized for this investigation. Second, all procedures were performed by a high-volume, fellowship-trained arthroplasty surgeon at a large, tertiary care institution. Therefore, our findings may not be generalizable to other practice settings. Third, though our institutional standard of care stipulates the routine collecting of PROMs for all patients, this was not possible in all cases. The response rate for questionnaires was > 70% in both cohorts, signifying a majority of data were successfully obtained. Fourth, patients in the RA-THA cohort reported lower baseline pre-operative HOOS-JR scores relative to patients in the mTHA cohort, which may have confounded the findings for this instrument. Lastly, the findings of our study are limited to a one-year time-horizon. Additional research is needed to better understand the terminal outcomes of patients who undergo surgery with these treatment strategies, as well as other issues like ceiling effects and joint-specific measures.

## Conclusions

The findings of this investigation demonstrated that the use of a novel, fluoroscopy-based RA-THA system resulted in greater improvements in HOOS scores relative to manual technique at one-year post-operative. The findings of this study represent the first PROMs-based investigation involving this system. Additional long-term studies that utilize an expanded cohort are needed to validate these findings.

### Electronic supplementary material

Below is the link to the electronic supplementary material.

Supplementary Material 1

## Data Availability

The data that support the findings of this study are available upon reasonable request from the corresponding author. The data are not publicly available due to privacy or ethical restrictions.
