# Supplementary material for: Use of a fluoroscopy-based robotic-assisted total hip arthroplasty system produced greater improvements in patient-reported outcomes at one year compared to manual, fluoroscopic-assisted technique
Source: Arch Orthop Trauma Surg. 2024 Feb 24;144(4):1843–50. doi: 10.1007/s00402-024-05230-8 (PMC10965579; doi:10.1007/s00402-024-05230-8)
Supplement: Supplementary file 1 — Supplementary Material 1 [file 402_2024_5230_MOESM1_ESM.docx]

Conflict of interest: Authors G.B.J.B., C.B.O., C.J.H., and L.S.SG. declare that they have no relevant competing interests. Authors C.A.D. and A.F.K. have the below disclosures:

- C.A.D.
  - Royalties: DePuySynthes, Mizuho-OSI
  - Speakers bureau: DePuySynthes, Medtronic
  - Stock or stock options: JointPoint, JointVue
- A.F.K.
  - Royalties: Innomed, ZimmerBiomet
  - Speakers bureau: ZimmerBiomet, Johnson and Johnson, BodyCad
  - Paid consultant: ZimmerBiomet, Johnson and Johnson, BodyCad, United Ortho, Orthopedic Development
  - Stock or stock options: ZimmerBiomet, Johnson and Johnson, Procter and Gamble
  - Research support: Signature Orthopedics
